# Interleukin-1 mediates ischaemic brain injury via distinct actions on endothelial cells and cholinergic neurons

**DOI:** 10.1016/j.bbi.2018.11.012

**Published:** 2019-02

**Authors:** Raymond Wong, Nikolett Lénárt, Laura Hill, Lauren Toms, Graham Coutts, Bernadett Martinecz, Eszter Császár, Gábor Nyiri, Athina Papaemmanouil, Ari Waisman, Werner Müller, Markus Schwaninger, Nancy Rothwell, Sheila Francis, Emmanuel Pinteaux, Adam Denés, Stuart M. Allan

**Affiliations:** a Faculty of Biology, Medicine and Health, University of Manchester, M13 9PT Manchester, UK; b “Momentum” Laboratory of Neuroimmunology, Institute of Experimental Medicine, Szigony u. 43, 1083 Budapest, Hungary; c Laboratory of Cerebral Cortex Research, Institute of Experimental Medicine, Szigony u. 43, 1083 Budapest, Hungary; d Institute for Molecular Medicine, University Medical Center of the Johannes Gutenberg University Mainz, 55131 Mainz, Germany; e Institute of Experimental and Clinical Pharmacology and Toxicology, University of Lübeck, 23538 Lübeck, Germany; f Department of Infection, Immunity & Cardiovascular Disease, Medical School, University of Sheffield, S10 2RX Sheffield, UK

## Abstract

The cytokine interleukin-1 (IL-1) is a key contributor to neuroinflammation and brain injury, yet mechanisms by which IL-1 triggers neuronal injury remain unknown. Here we induced conditional deletion of IL-1R1 in brain endothelial cells, neurons and blood cells to assess site-specific IL-1 actions in a model of cerebral ischaemia in mice. Tamoxifen treatment of IL-1R1 floxed (^fl/fl^) mice crossed with mice expressing tamoxifen-inducible Cre-recombinase under the Slco1c1 promoter resulted in brain endothelium-specific deletion of IL-1R1 and a significant decrease in infarct size (29%), blood-brain barrier (BBB) breakdown (53%) and neurological deficit (40%) compared to vehicle-treated or control (IL-1R1^fl/fl^) mice. Absence of brain endothelial IL-1 signalling improved cerebral blood flow, followed by reduced neutrophil infiltration and vascular activation 24 h after brain injury. Conditional IL-1R1 deletion in neurons using tamoxifen inducible nestin-Cre mice resulted in reduced neuronal injury (25%) and altered microglia-neuron interactions, without affecting cerebral perfusion or vascular activation. Deletion of IL-1R1 specifically in cholinergic neurons reduced infarct size, brain oedema and improved functional outcome. Ubiquitous deletion of IL-1R1 had no effect on brain injury, suggesting beneficial compensatory mechanisms on other cells against the detrimental effects of IL-1 on endothelial cells and neurons. We also show that IL-1R1 signalling deletion in platelets or myeloid cells does not contribute to brain injury after experimental stroke. Thus, brain endothelial and neuronal (cholinergic) IL-1R1 mediate detrimental actions of IL-1 in the brain in ischaemic stroke. Cell-specific targeting of IL-1R1 in the brain could therefore have therapeutic benefits in stroke and other cerebrovascular diseases.

## Highlights

• Brain endothelial cells mediate detrimental actions of IL-1 in cerebral ischemia.
• Neuronal cholinergic IL-1R1 also mediate detrimental actions of IL-1 in brain injury.
• Brain endothelial IL-1 actions reduce cortical perfusion after cerebral ischemia.
• Ubiquitous IL-1R1 deletion does not affect injury, suggesting compensatory mechanisms.

## 1 Background

Inflammation is a major contributor to stroke pathophysiology and is therefore an attractive therapeutic target. A key mediator of inflammation is the pro-inflammatory cytokine interleukin-1 (IL-1) (Dinarello et al., 2012). IL-1, expressed as two isoforms IL-1α and IL-1β, is upregulated rapidly after experimental cerebral ischaemia and very early expression is thought to occur in monocyte and macrophage lineages, whilst slightly delayed expression occurs in astrocytes, neurons, endothelial cells and invading immune cells (Ching et al., 2005, Pinteaux et al., 2009, Denes et al., 2011). Preclinical studies using experimental animal models have demonstrated the importance of IL-1 in stroke. Central or systemic administration of exogenous recombinant IL-1β in rodents subjected to middle cerebral artery occlusion (MCAo) exacerbates brain damage (Yamasaki et al., 1995, Stroemer and Rothwell, 1998, McColl et al., 2007), whilst disruption of both IL-1α and IL-1β in IL-1α/β knockout (KO) mice results in a 70% reduction in infarct volume (Boutin et al., 2001). There is extensive experimental evidence showing that blockade of IL-1 signalling using the IL-1 receptor antagonist (IL-1Ra) is protective in stroke and other forms of brain injury, and early stage clinical trials of IL-1Ra in both ischaemic and haemorrhagic stroke have to date shown potentially promising results (Sobowale et al., 2016). However, the cellular mechanisms by which IL-1 mediates brain injury following cerebral ischaemia remain unknown.

IL-1 is known to exert its actions via binding and activation of its main functional IL-1 type 1 receptor (IL-1R1) (Sims et al., 1988). IL-1R1 is expressed on the cerebrovasculature (Konsman et al., 2004) and *in vitro* studies also suggest that IL-1 acts in the brain through endothelial cells (Thornton et al., 2010, Summers et al., 2013), whilst toxic actions of IL-1 are mediated via cerebrovascular activation and transmigration of neutrophils (Allen et al., 2012) *in vitro*. The knockdown of endothelial IL-1R1 has been investigated *in vivo* (Li et al., 2011, Ching et al., 2007) but has been focussed on ubiquitous knockdown rather than inhibiting specific endothelial cell subsets, such as in the brain. IL-1 acts both peripherally and centrally (Denes et al., 2013) but precise brain specific actions have not yet been identified.

IL-1 also has diverse actions on neurons, including fast electrophysiological firing (Diem et al., 2003, Desson and Ferguson, 2003), potentiation of excitotoxicity and changes in neuronal gene expression (Denes et al., 2011, Tsakiri et al., 2008). However, functional data showing the effect of IL-1 on neurons and endothelial cells have been obtained only from *in vitro* studies (Lazovic et al., 2005, Andre et al., 2006), including our previous work showing IL-1 acts on neurons to produce inflammatory mediators (Tsakiri et al., 2008), suggesting neuronal signalling could contribute to detrimental neuroinflammatory responses, though the contribution of cell specific IL-1 actions to brain injury *in vivo* remains unknown.

The objective of this study was to determine the target cells of IL-1 action during ischaemic brain injury in mice. Tools to selectively and conditionally delete IL-1R1 in different cell types *in vivo* have become available recently (Abdulaal et al., 2016, Bruttger et al., 2015). Thus, we investigated the contribution of endothelial cells and neurons to ischaemic brain injury by deleting IL-1R1 from brain endothelial cell or neurons (including cholinergic neuronal cells). We also assessed the effects of IL-1R1 deficiency in platelets and myeloid cells, cell types that are known to contribute to diverse forms of brain injury and that are involved in systemic IL-1 actions (Thornton et al., 2010, Denes et al., 2011, Iadecola and Anrather, 2011). We show that both brain endothelial and neuronal IL-1R1 mediate the actions of IL-1 on brain injury via functionally distinct mechanisms, some of which (i.e. effects on cerebral perfusion) are apparent in the first hour after the ischaemic insult. We also reveal that IL-1R1 on cholinergic neurons themselves represent a potential therapeutic target against the detrimental effects of IL-1 after acute brain injury.

## 2 Materials and methods

### 2.1 Experimental design

Animal procedures were carried out in accordance with the Animal Scientific Procedures Act (1986) and the European Council Directive 2010/63/EU, and were approved by the Animal Welfare and Ethical Review Body, University of Manchester, UK and the Animal Care and Use Committee of the Institute of Experimental Medicine, Budapest, Hungary. Experiments followed ARRIVE (Kilkenny et al., 2010) and IMPROVE guidelines (Percie du Sert, 2017). A code was allocated to each animal by a non-experimenter and was randomly assigned to different treatment groups. During all surgical procedures and functional tests the experimenter was blinded to treatment.

### 2.2 Animals

**Fig. 1:**
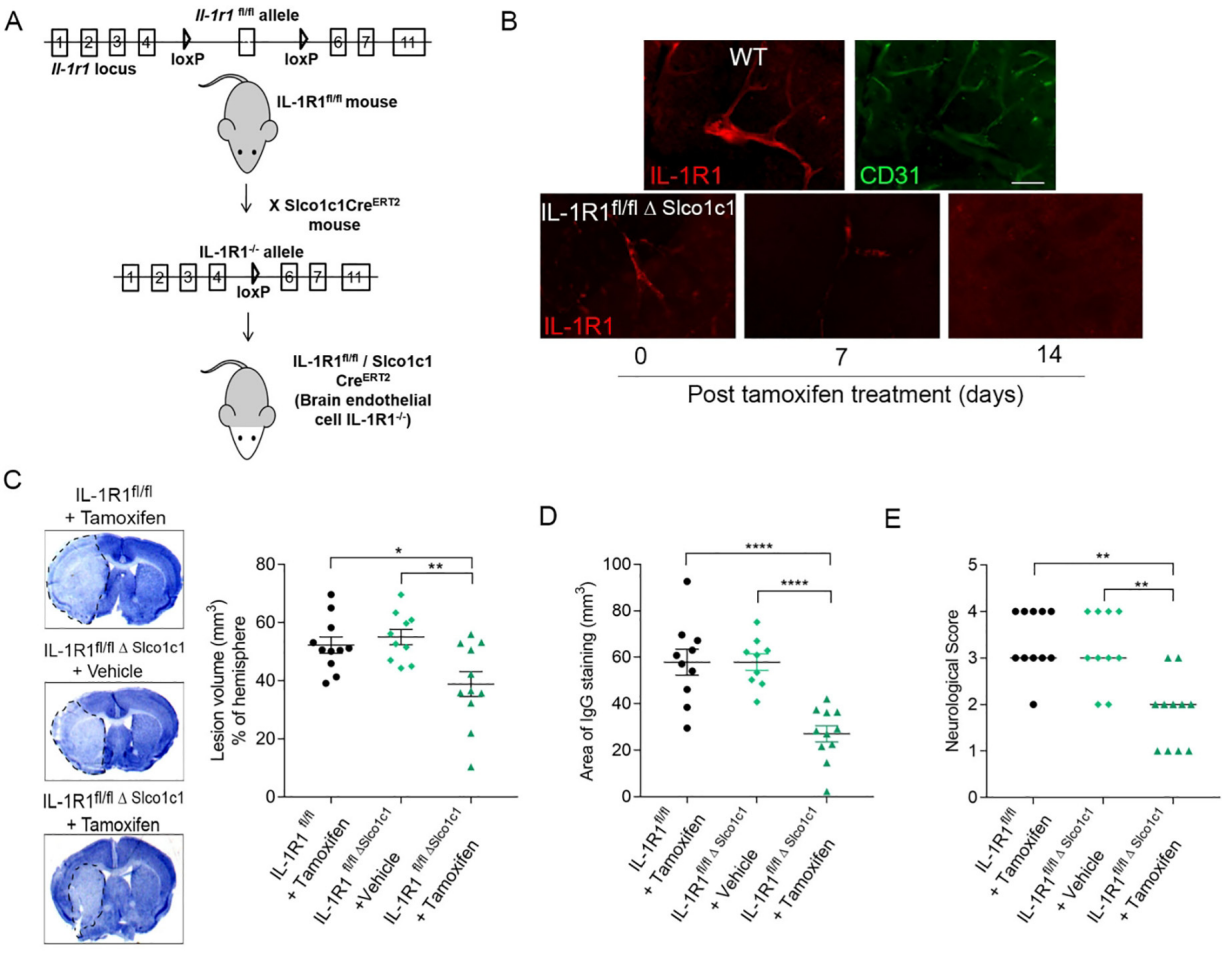
Brain endothelial IL-1R1 deletion reduces infarct volume and BBB permeability and improves neurological function. IL-1R1^fl/fl Δ Slco1c1^ (Brain endothelial-specific IL-1R1) mice were generated by crossing IL-1R1^fl/fl^ mice (exon 5 of the *Il1r1* gene flanked with loxP sites) with Slco1c1 Cre^ERT2^ mice (expressing Cre recombinase under the promoter of the thyroxine transporter (A). IL-1R1 protein expression was reduced over time in blood vessels identified by CD31 immunostaining in IL-1R1^fl/fl Δ Slco1c1^ animals after tamoxifen treatment to total deletion by day 14 (B). IL-1R1 deletion in brain endothelial cells (IL-1R1^fl/fl Δ Slco1c1^ mice treated with tamoxifen) reduced infarct volume as assessed on cresyl violet stained brain sections (C), IgG leakage (D) and improved neurological function (E) compared to control group (IL-1R1^fl/fl Δ Slco1c1^ mice treated with vehicle). IL-1R1^fl/fl Δ Slco1c1^ mice treated with vehicle; n = 10, IL-1R1^fl/fl Δ Slco1c1^ mice treated with tamoxifen; n = 11, IL-1R1^fl/fl^ treated with tamoxifen; n = 11. ^*^p ≤ 0.05, ^**^p ≤ 0.01, ^***^p ≤ 0.001, ^****^p ≤ 0.0001. All parametric data are expressed as means ± SEM, medians are shown in the case of neurological scores. (For interpretation of the references to colour in this figure legend, the reader is referred to the web version of this article.)

All mice were on a C57BL/6J background and only males were used. Brain endothelial-specific IL-1R1 knockout (KO) mice were generated by crossing mice in which exon 5 of the *Il1r1* gene is flanked with loxP sites [IL-1R1 floxed (^fl/fl^)] (Abdulaal et al., 2016) with mice expressing Cre recombinase under the promoter of the thyroxine transporter (Slco1c1) that is specifically expressed in brain endothelial cells (Ridder et al., 2011) (thereafter named IL-1R1^fl/fl Δ Slco1c1^) (Fig. 1A). Brain endothelial IL-1R1 deletion was achieved by injection of tamoxifen (2 mg/100 µl in corn oil, Sigma-Aldrich) for five consecutive days in 6–9 week old male mice. Controls were IL-1R1^fl/fl Δ Slco1c1^ male mice injected with vehicle (corn oil) as well as IL-1R1^fl/fl^ mice treated with tamoxifen. Mice allocated for the detection of IL-1R1 expression were culled 0, 7 and 14 days after tamoxifen administration, whilst mice allocated for experimental stroke underwent surgery at 21 days after the start of tamoxifen or vehicle administration. Tamoxifen inducible nestin-Cre mice were crossed with IL-1R1^fl/fl^ mice to delete IL-1R1 in neurons following two consecutive administrations of 2 mg/100 μl tamoxifen (48 h apart) in 2–4 week old male mice (thereafter named IL-1R1^fl/fl Δ Nestin^). Ubiquitous IL-1R1 KO mice (named IL-1R1^−/−^) were generated by crossing IL-1R1^fl/fl^ mice with K14-Cre mice, as previously reported (Couzin-Frankel, 2012). To delete IL-1R1 selectively from cholinergic neurons, IL-1R1^fl/fl^ mice were crossed with ChAT-cre mice (IL-1R1^fl/fl Δ ChAT^). To selectively and constitutively delete IL-1R1 in myeloid cells or platelets, IL-1R1^fl/fl^ mice were crossed with LysM-Cre mice (IL-1R1^fl/fl Δ LysM^) or PF4-Cre mice (IL-1R1^fl/fl Δ PF4^), respectively. All animals were maintained at 21 ± 1 °C, 55 ± 10% humidity, in a 12 h light-dark cycle with free access to food and water.

In total, 10 IL-1R1^fl/fl Δ Slco1c1^ animals injected with vehicle, 11 IL-1R1^fl/fl Δ Slco1c1^ animals injected with tamoxifen and 11 IL-1R1^fl/fl^ animals injected with tamoxifen, were used in this study to determine the effect of brain endothelial cell IL-1R1 deletion; 10 IL-1R1^fl/fl^ and 10 tamoxifen-treated IL-1R1^fl/fl Δ Nestin^ were used to assess the effect of neuronal IL-1R1 deletion; 13 IL-1R1^fl/fl^ mice and 10 IL-1R1^−/−^ mice were used to determine the effect of ubiquitous IL-1R1 deletion; 9 IL-1R1^fl/fl Δ ChAT^ mice, 10 IL-1R1^fl/fl^ mice, 14 IL-1R1^fl/fl^ mice, 14 IL-1R1 ^fl/fl Δ PF4^ and 12 IL-1R1 ^fl/fl Δ LysM^ mice were used to assess the effect of IL-1R1 deletion in cholinergic neurons, platelets or myeloid cells, respectively. The pre-determined inclusion criteria for analysis were as follows; decline in Doppler signal of at least 70%, no subarachnoid haemorrhages and survival to 24 h. Subarachnoid haemorrhage was identified post-mortem by the presence of excessive bleeding on the external surface of the brain, typically close to the filament location. Animals excluded from analysis due to early mortality included; two IL-1R1^fl/fl Δ Slco1c1^ mice treated with vehicle and two with tamoxifen, four IL-1R1^fl/fl^, one IL-1R1^fl/fl Δ Nestin^ and two IL-1R1^fl/fl Δ ChAT^ animal due to surgical artefacts, whilst a single IL-1R1^fl/fl Δ Slco1c1^ vehicle-treated animal was culled early for welfare. Exclusions for cerebral haemorrhages included; two IL-1R1^fl/fl Δ Slco1c1^ mice treated with vehicle, three with tamoxifen, one IL-1R1^fl/fl^ animal treated with tamoxifen and one IL-1R1^fl/fl Δ Nestin^ animal. Four additional mice were excluded from analysis pre hoc based on the criteria detailed above in that an insufficient drop (>70%) in cerebral blood flow was observed. In total there were 23 mice excluded from the study, which is 14.6% of the total used.

### 2.3 Focal cerebral ischaemia

Anaesthesia was induced by inhalation of 4% isoflurane (30% oxygen and 70% nitrous oxide gas mix, AbbVie Ltd, UK or Linde Ltd, Hungary) and was maintained at 1.75%. Body temperature was monitored throughout surgery (via rectal probe) and maintained at 37 °C ± 0.5 °C using a heating blanket (Harvard Apparatus, Edenbridge, Kent, UK). A laser Doppler blood flow monitor (Oxford Optronix, Abingdon, UK) was used to monitor cerebral blood flow (CBF). Focal cerebral ischaemia was induced by MCAo based on a previously described protocol (Wong et al., 2014). Briefly, a hole was made into the temporalis muscle (6 mm lateral and 2 mm posterior from bregma) to allow a 0.5 mm diameter flexible laser-Doppler probe to be fixed onto the skull and secured in place by tissue adhesive (Vetbond, UK). A midline incision was made on the ventral surface of the neck and the right common carotid artery isolated and ligated. Topical anaesthetic (EMLA, 5% prilocaine and lidocaine, AstraZeneca, UK) was applied to skin incision sites prior to incision. The internal carotid artery and the pterygopalatine artery were temporarily ligated. A 6-0 monofilament (Doccol, Sharon, MA, USA) was introduced into the internal carotid artery via an incision in the common carotid artery (endothelial-specific IL-1R1 deletion study) or via the external carotid artery (ubiquitous, neuronal-specific-, myeloid cell-specific and platelet-specific IL-1R1 deletion studies). The filament was advanced approximately 10 mm distal to the carotid bifurcation, beyond the origin of the middle cerebral artery. Relative CBF was monitored for the first 30–45 min following MCAo, during which time relative CBF had to reduce by at least 70% of pre-ischaemic values for inclusion. After 30 min of occlusion (endothelial-specific IL-1R1 deletion study) or 45 min of occlusion (ubiquitous, neuronal-specific-, myeloid cell-specific and platelet-specific IL-1R1 deletion studies) the filament was withdrawn back into the common carotid artery to allow reperfusion to take place. The wound was sutured and mice received a subcutaneous bolus dose of saline for hydration (500 µl) and a general analgesic (Buprenorphine, 0.05 mg/kg injected subcutaneously, Vetergesic, UK). Animals were kept at 26–28 °C until they recovered from anaesthesia and surgery, before being transferred back to ventilated cages suspended over a heating pad with free access to mashed food and water in normal housing conditions.

### 2.4 Tissue processing

Animals were perfused transcardially with 0.9% saline followed by 4% paraformaldehyde (PFA) under either terminal 3% isoflurane (30% oxygen and 70% nitrous oxide gas mix) or ketamine-xylazine anaesthesia. Brains were then removed and left to post-fix for 24 h either in 4% PFA or in 10% sucrose 4% PFA before being transferred either to 30% sucrose or to 10% sucrose-PBS. After 24 h in 10% or 30% sucrose, brains were sectioned into 25–30 µm coronal sections using a sledge microtome (Bright, Cambridgeshire, UK or Leica, Germany) for subsequent Cresyl violet staining and immunohistochemistry.

### 2.5 Functional outcomes

Animals were assessed for neurological deficits 24 h after MCAo a using a 5-point scoring system (Iadecola et al., 1997). The neurological scores were as follows: 0, normal motor function; 1, flexion of torso and contralateral forelimb when mouse was lifted by the tail; 2, circling to the contralateral side when mouse is held by the tail on a flat surface, but normal posture at rest; 3, leaning to the contralateral side at rest, 4, no spontaneous motor activity. In IL-1R1^fl/fl Δ ChAT^ mice functional outcome has also been assessed by a composite neurological score optimized for mice to obtain a more comprehensive readout (Orsini et al., 2012, Clark et al., 1997). Composite scores range from 0 (healthy mice) to 56 (worst performance) by adding up scores from 13 categories as follows: hair (0–2), ears (0–2), eyes (0–4), posture (0–4), spontaneous activity (0–4), and epileptic behavior (0–12), and focal deficits: body symmetry (0–4), gait (0–4), climbing on a surface held at 45° (0–4), circling behavior (0–4), front limb symmetry (0–4), compulsory circling (0–4), and whisker response to a light touch (0–4). Results are expressed as composite neurological score.

### 2.6 Laser speckle contrast imaging (LSCI)

At the end of the MCAo surgery, mice were transferred to the stereotaxic frame and LSCI measurements performed 30 min after reperfusion under isoflurane anaesthesia, using a PeriCam PSI High Resolution system (Perimed AB, Järfälla-Stockholm, Sweden). To assess CBF changes in different groups of mice in a uniform manner, tamoxifen-treated IL-1R1^fl/fl Δ Slco1c1^ mice and IL-1R1^fl/fl Δ Nestin^ mice together with tamoxifen-treated control IL-1R1^fl/fl^ mice were subjected to MCAo for 45 min (left side occluded). LSCI measurements were performed 30 min after reperfusion. The skin on the top of the skull was opened and imaging performed through the intact skull bone to visualize cortical perfusion changes (Winship, 2014) for 10 min at 16 frames/sec in 20 µm/pixel resolution, using a 10 × 10 mm field of view. To evaluate recovery of blood flow in the penumbra after stroke, perfusion changes were assessed in three adjacent regions of interest (ROI) in the primary MCA area (Fig. 5A). LSCI is particularly sensitive to assess perfusion changes in the microcirculation. The area of cortical sinusoids have been excluded from ROIs and only measurements without motion artefacts have been analysed to minimize bias (Eriksson et al., 2014, Dunn, 2012). Area under the curve (AUC) values over the 10 min imaging period for each ROI were determined and data expressed as percentage values of the corresponding contralateral ROI.

### 2.7 Histology and immunohistochemistry

Lesion volumes were measured using Cresyl violet staining, as previously described (McColl et al., 2007). For each brain, infarcts were measured on defined coronal sections (using image J), spaced approximately 360 µm apart. Each defined coronal section, with its brain co-ordinates and lesion was integrated to estimate total lesion volume for each brain and corrected for oedema.

Blood-brain-barrier (BBB) permeability was assessed by peroxidase-based immunohistochemistry for circulating IgG infiltration in the brain. Endogenous peroxidase activity (0.3% H_2_0_2_ in dH_2_O for 10 min) and non-specific staining [5% normal horse serum, 0.3% triton in phosphate buffered saline (PBS) for 10 min] were blocked, and sections incubated in biotinylated anti-mouse IgG antibody (1:500, #BA-2000, Vector Laboratories, UK) for 2 h. Sections were then washed and incubated with avidin–biotin–peroxidase complex (1:500, Vectastain Elite ABC HRP Kit, #PK-6100, Vector Laboratories, UK), and detected by colorimetry using a diaminobenzidine (DAB) solution (0.05% DAB, 0.005% H_2_O_2_ in dH_2_O). Sections were left to dry, before being mounted using DPX mounting medium (Fisher Scientific, UK), cover slipped and imaged using an Olympus BX51 upright microscope with Coolsnap ES camera (Photometrics, UK) for image capture. The total IgG volume for the brain was determined by measuring IgG on individual sections and integrating them to determine total IgG volume, as described earlier for lesion volume.

### 2.8 Immunofluorescence

Brain sections were incubated with blocking buffer consisting of 5% normal donkey serum (Jackson laboratories, Bar Harbor, ME, USA), 1% bovine serum albumin (BSA), 0.1% Triton X-100, 0.05% Tween 20 (Sigma-Aldrich), 0.2 M Glycine (Fisher Scientific) in PBS for 1 h. Blocking buffer was then removed and sections were incubated with primary antibody, diluted in primary antibody buffer (1% BSA, 0.3% Triton X-100 in PBS), with Lectin (1:100, Lycopersicon esculentum, #L0651, Sigma-Aldrich) at 4 °C overnight. Primary antibodies used in this study included; goat anti-IL-1R1 (1:100, #AF771, R & D Systems, UK), sheep anti-VWF (1:100, Abcam, UK), rabbit anti-Iba1 (1:1000, #019–19741, Wako, USA), goat ant-intracellular adhesion molecule 1 (ICAM-1, 1:200, #AF796, R&D Systems, UK), goat anti-vascular cell adhesion protein 1 (VCAM-1, 1:100, R&D Systems, UK), SJC4 (1:10,000, kindly gifted by Professor Daniel Anthony, University of Oxford, UK), rat anti-CD45 (1:250, #MCA1388, AbD Serotec, UK), goat-anti MPO (1:250, #AF3667, Novus Biologicals, UK), mouse anti-neurofilament-H (1:500, #SMI-32P, Covance, USA), rabbit anti-P2RY12 (1:500, #55043AS AnaSpec, Belgium), mouse anti-Cre recombinase (1:500, #MAB3120, Millipore, Germany) and chicken-anti PGP9.5 (1:500, #ab72910, Abcam, UK), rabbit anti- Annexin V (1:250, #NB100-1930, Novus Biologicals, UK) and mouse-anti NeuN (1:500, #MAB377, Millipore, Germany). Sections were washed in 0.1% Tween in PBS, and incubated with secondary antibodies diluted in 0.05% tween in PBS. Secondary antibodies consisted of Alexa-Fluor 488 (1:500 rabbit, goat, ThermoFisher, UK), Alexa-Fluor 594 (1:500, sheep, goat, ThermoFisher, UK) and Alexa-Fluor 350 (1:100, streptavidin, ThermoFisher, UK), donkey anti-rabbit A488 (1:500, # A21206, Invitrogen, USA), streptavidin DyL405 (1:500, #016-470-084, Jackson ImmunoResearch, USA), donkey anti-goat A488 (1:500, #A11055, Invitrogen, USA), donkey anti-rat A594 (1:500, #A21209, Invitrogen, USA) and donkey anti-rabbit A647 (1:500, #711-605-152, Jackson ImmunoResearch, USA). After a 2 h incubation, secondary antibodies were removed, and sections were washed with 0.1% Tween in PBS, mounted on glass slides and left to dry in the dark, before being cover slipped with mounting medium (ProLong Gold without DAPI, ThermoFisher, UK).

### 2.9 Image analysis

Analyses for immunohistochemistry were conducted on coronal sections taken at the same co-ordinates (approximately +1 mm anterior to bregma), and both ipsilateral and contralateral hemispheres (to the side of the brain lesion) regions were measured in each animal. Neutrophils were counted in the middle of the striatum and in the cerebral cortex. Analysis of vascular markers (ICAM-1, VCAM-1) and Iba1 immunostaining was conducted in the penumbra region of the cortex and in the striatum. The activation states of Iba1-positive microglia were scored based on the morphology, as described previously (40), or numbers of activated microglia counted based on the levels of Iba1 and CD45 expression and morphology. Briefly, scoring was as follows: 0, Resting/Ramified; 1, De-ramifying/Re-ramifying; 2, Activated/Amoeboid; 3, Clustered & activated. Image analysis was carried out blinded to the experimental group status on three randomly selected ROI/brain region/slice on three coronal slices/animal.

### 2.10 Statistical analysis

Sample size for experimental stroke studies were determined by a priori power calculation using G*Power 3.1.9.2 with mean differences and standard deviations based on pilot studies and previous experiments (power 80%, α 0.05). Data were assessed for normal distribution using the Shapiro-Wilk W-test in order to determine parametric or non-parametric analysis. Lesion volume and BBB breakdown were analysed with one-way ANOVA followed by post-hoc Tukey multiple comparisons between treatments groups. Mann-Whitney test was used to analyse differences between control and IL-1R1^fl/fl Δ ChAT^ mice due to the lack of normality of data sets. Neurological scoring was analysed with non-parametric Mann-Whitney test (2 groups) or Kruskal-Wallis test combined with Dunn’s multiple comparisons test. Immunofluorescence data were analysed by two-way ANOVA, and post-hoc Tukey’s multiple comparisons between matched hemisphere and treatment groups. Significant difference was considered for p < 0.05.

## 3 Results

### 3.1 Brain endothelial IL-1R1 deletion reduces infarct volume, BBB permeability and improves neurological function

First, we investigated brain injury after targeted deletion of IL-1R1 from the cerebrovasculature. IL-1R1^fl/fl Δ Slco1c1^ animals (Fig. 1A) injected with tamoxifen showed reduced IL-1R1 protein expression in cerebral blood vessels at day 7 and IL-1R1 expression was completely ablated on day 14 (Fig. 1B). Experimental animals in this study were used at day 21 onwards.

Deletion of IL-1R1 in brain endothelial cells (IL-1R1^fl/fl Δ Slco1c1^ mice) significantly reduced both ischaemic infarct (29% decrease, Fig. 1C, 95% CI: 4.33 to 27.99, p = 0.0058; F (2, 29) = 6.67) and IgG leakage (53% decrease, Fig. 1D, 95% CI: 15.27 to 46.47, p < 0.0001; F (2, 27) = 17.82) compared to vehicle-treated IL-1R1^fl/fl Δ Slco1c1^ animals, whilst IL-1R1^fl/fl^ animals treated with tamoxifen had infarcts and BBB injury based on IgG leakage comparable to vehicle-treated IL-1R1^fl/fl Δ Slco1c1^ mice (Fig. 1C and D). IL-1R1 deletion in brain endothelial cells resulted in reduced neurological deficits (40% decrease, Fig. 1E, p < 0.0065) compared to vehicle-treated IL-1R1^fl/fl Δ Slco1c1^ mice, with experimental groups correlating similarly to infarct volume and BBB disruption.

### 3.2 Brain endothelial IL-1R1 deletion reduces cerebrovascular activation and neutrophil migration

**Fig. 2:**
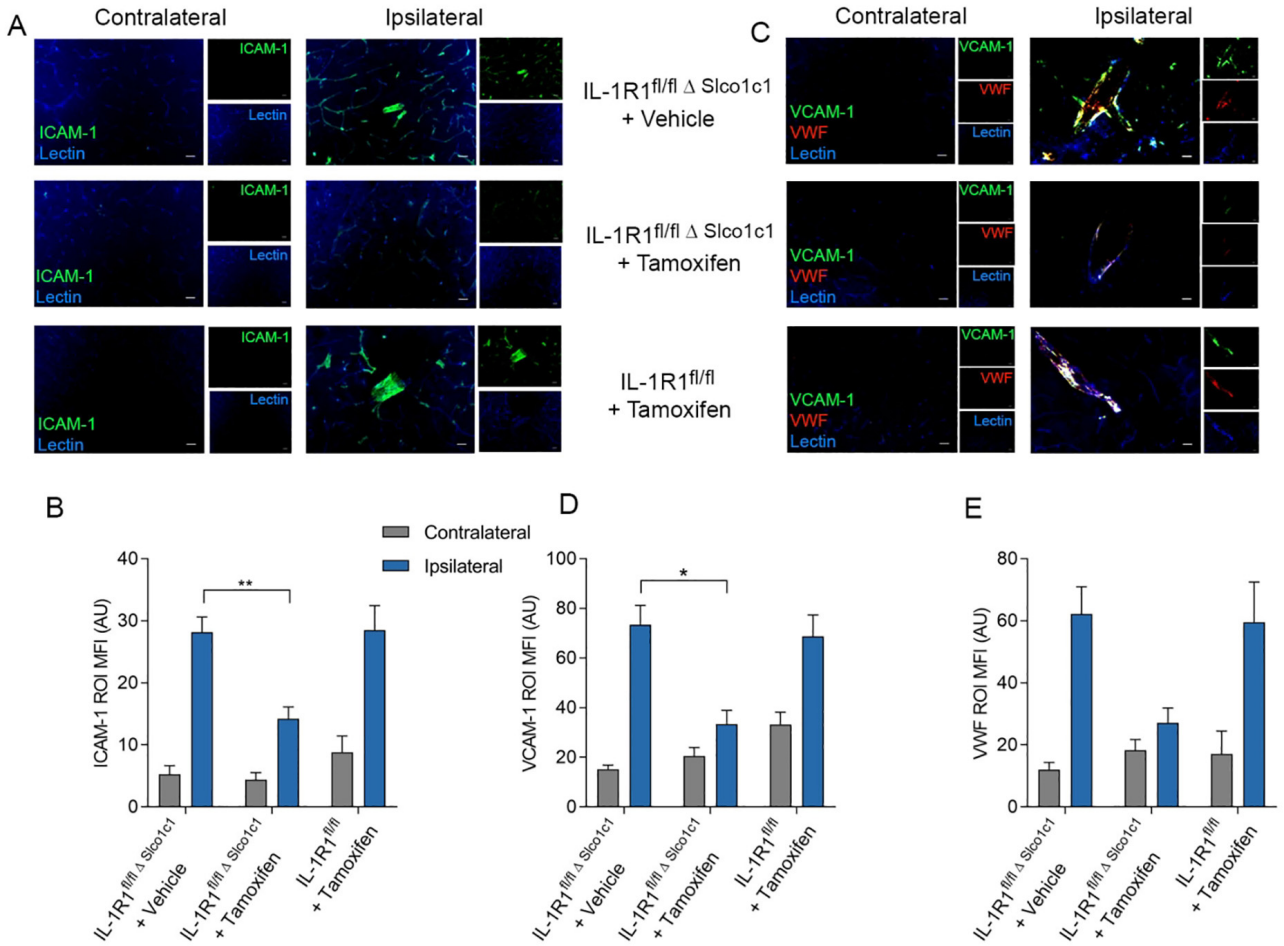
Brain endothelial IL-1R1 deletion reduces vascular activation. Immunostaining with ICAM-1 (co-stained with lectin to identify cerebral blood vessels) showed increased ICAM-1 cerebrovascular staining in the ipsilateral hemisphere when compared to the contralateral side 24 h after MCAo (A), and deletion of IL-1R1 on the brain endothelium decreased ICAM-1 expression (IL-1R1^fl/fl Δ Slco1c1^ mice treated with tamoxifen) in comparison to control (IL-1R1^fl/fl Δ Slco1c1^ mice treated with vehicle) (B). VCAM-1 Immunostaining (co-stained with lectin to identify cerebral blood vessels and VWF for endothelial cell activation) showed increased VCAM-1 cerebrovascular staining in the ipsilateral hemisphere when compared to the contralateral side after stroke (C), and deletion of IL-1R1 on the brain endothelium decreased VCAM-1 expression (IL-1R1^fl/fl Δ Slco1c1^ mice treated with tamoxifen) in comparison to control (IL-1R1^fl/fl Δ Slco1c1^ mice treated with vehicle) (D). No change in the number of VWF-positive blood vessels was seen (E). IL-1R1^fl/fl Δ Slco1c1^ mice treated with vehicle; n = 10, IL-1R1^fl/fl Δ Slco1c1^ mice treated with tamoxifen; n = 11, IL-1R1^fl/fl^ treated with tamoxifen; n = 7. ^*^p ≤ 0.05, ^**^p ≤ 0.01. Region of interest MFI measured in arbitrary units (AU). Data are expressed as means ± SEM.

As reported previously (Wang et al., 1994), MCAo increased cerebral vascular expression of ICAM-1 (Fig. 2A and B) and VCAM-1 (Fig. 2C and D) in the ipsilateral hemisphere, compared to the contralateral hemisphere [F (1, 25) = 113.8, p ≤ 0.001; F (1, 25) = 98.82, p ≤ 0.001, respectively]. However, brain endothelial IL-1R1 deletion reduced cerebrovascular activation in IL-1R1^fl/fl Δ Slco1c1^ mice by 50% (95% CI: 1.86 to 12.95, p = 0.007) for ICAM-1 (Fig. 2B) and 55% (95% CI: 1.81–33.08, p = 0.027) for VCAM-1 (Fig. 2D) compared to vehicle-treated animals [F (2, 25) = 8.919, p = 0.0012; F (2, 25) = 7.043, p = 0.0038, respectively]. Cerebral activation of VWF was reduced by 56% though this was not significant (95% CI: −4.71 to 33.42, p = 0.1668; F (2, 25) = 2.412, p = 11.02). Tamoxifen-treated IL-1R1^fl/fl^ mice had equivalent levels of ICAM-1 (Fig. 2B) and VCAM-1 (Fig. 2D) to vehicle-treated IL-1R1^fl/fl Δ Slco1c1^ mice.

We have demonstrated previously that IL-1 actions on brain endothelial cells *in vitro* contribute to neutrophil infiltration and subsequent neurotoxicity (Allen et al., 2012). Since our data demonstrate that brain endothelial IL-1R1 contributes to brain damage and BBB dysfunction, we next investigated the possible mechanisms underlying brain endothelial IL-1R1 deletion-induced ischaemic damage, by assessing stroke induced neutrophil infiltration after IL-1R1 deletion.

**Fig. 3:**
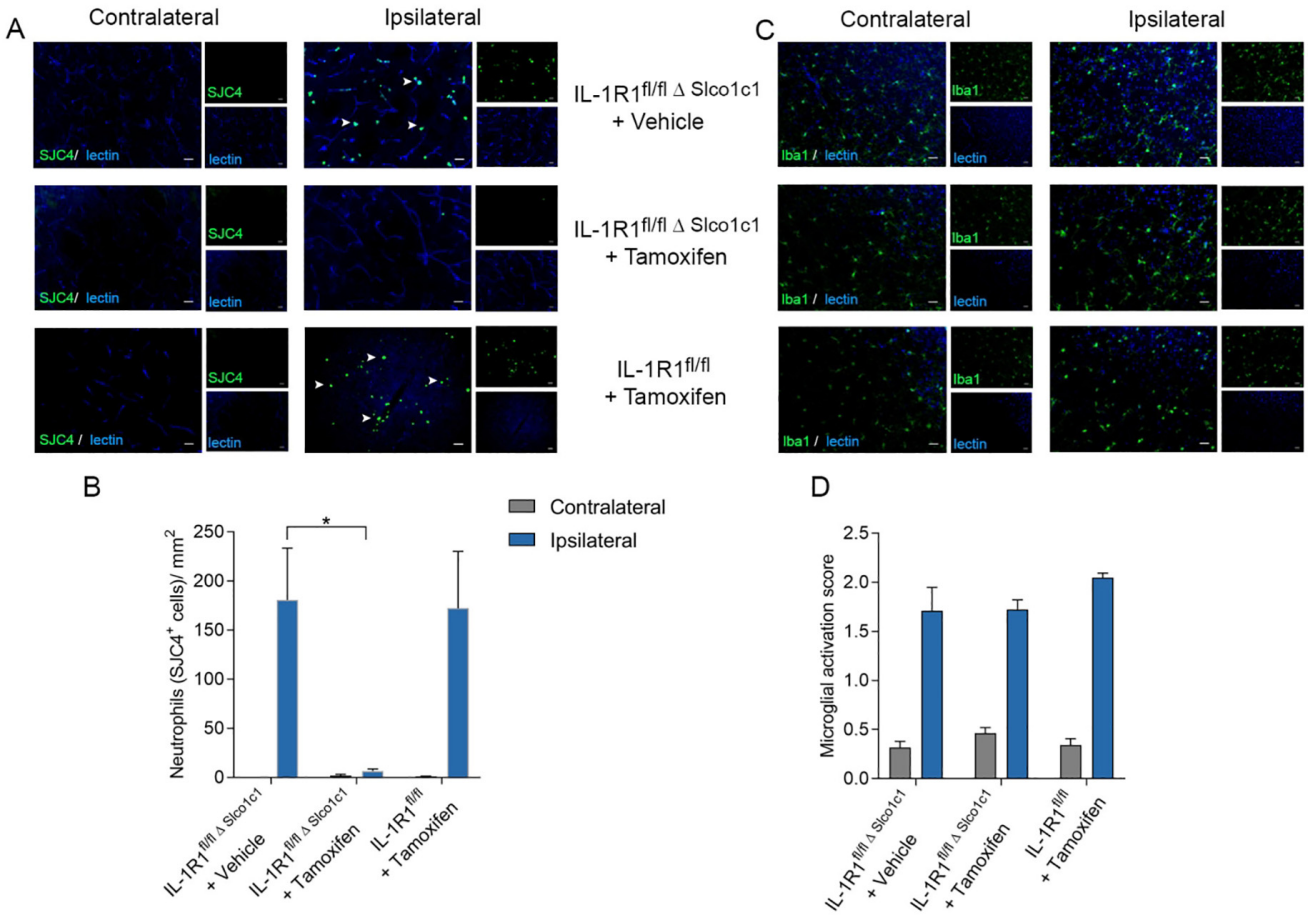
Brain endothelial IL-1R1 deletion reduces neutrophil cerebrovascular migration but does not influence microglial activation. Immunostaining for neutrophils using SCJ4 antibody (co-stained with lectin to identify cerebral blood vessels) reveals increased number of neutrophils in the ipsilateral hemisphere 24 h after MCAo in comparison to the contralateral side (A), and deletion of IL-1R1 on the brain endothelium reduced the number of neutrophils (IL-1R1^fl/fl Δ Slco1c1^ mice treated with tamoxifen) in comparison to control (IL-1R1^fl/fl Δ Slco1c1^ mice treated with vehicle) (B). Immunostaining for microglia using Iba1 (co-stained with lectin to identify cerebral blood vessels) revealed no differences in cell number (C), but did show increased cell activation in the damaged region after stroke. However, no differences in activation states were found between experimental groups (D). IL-1R1^fl/fl Δ Slco1c1^ mice treated with vehicle; n = 10, IL-1R1^fl/fl Δ Slco1c1^ mice treated with tamoxifen; n = 11, IL-1R1^fl/fl^ treated with tamoxifen; n = 7. ^*^p ≤ 0.05. Data are expressed as means ± SEM.

After MCAo, neutrophils migrate to the area of infarct, in significantly increased numbers compared to the undamaged contralateral side (Fig. 3A). IL-1R1^fl/fl Δ Slco1c1^ mice treated with tamoxifen, showed almost a complete loss (94% reduction, p = 0.0114) of neutrophil migration compared to vehicle treatment (95% CI: 17.95 to 154.4, p = 0.0114; F (2, 25) = 6.093), whilst IL-1R1^fl/fl^ animals treated with tamoxifen had similar neutrophil numbers compared to vehicle-treated IL-1R1^fl/fl Δ Slco1c1^ animals (Fig. 3B). Although IL-1R1 deletion on the brain endothelium dramatically reduced neutrophil migration induced by cerebral ischaemia, there was no effect on microglial activation, and the number of microglia were unchanged (data not shown) compared to vehicle-treated IL-1R1^fl/fl Δ Slco1c1^ or IL-1R1^fl/fl^ control animals after MCAo (Fig. 3C and D).

### 3.3 Neuronal IL-1R1 deletion reduces infarct volume and leads to altered microglia-neuron interactions in the penumbra

**Fig. 4:**
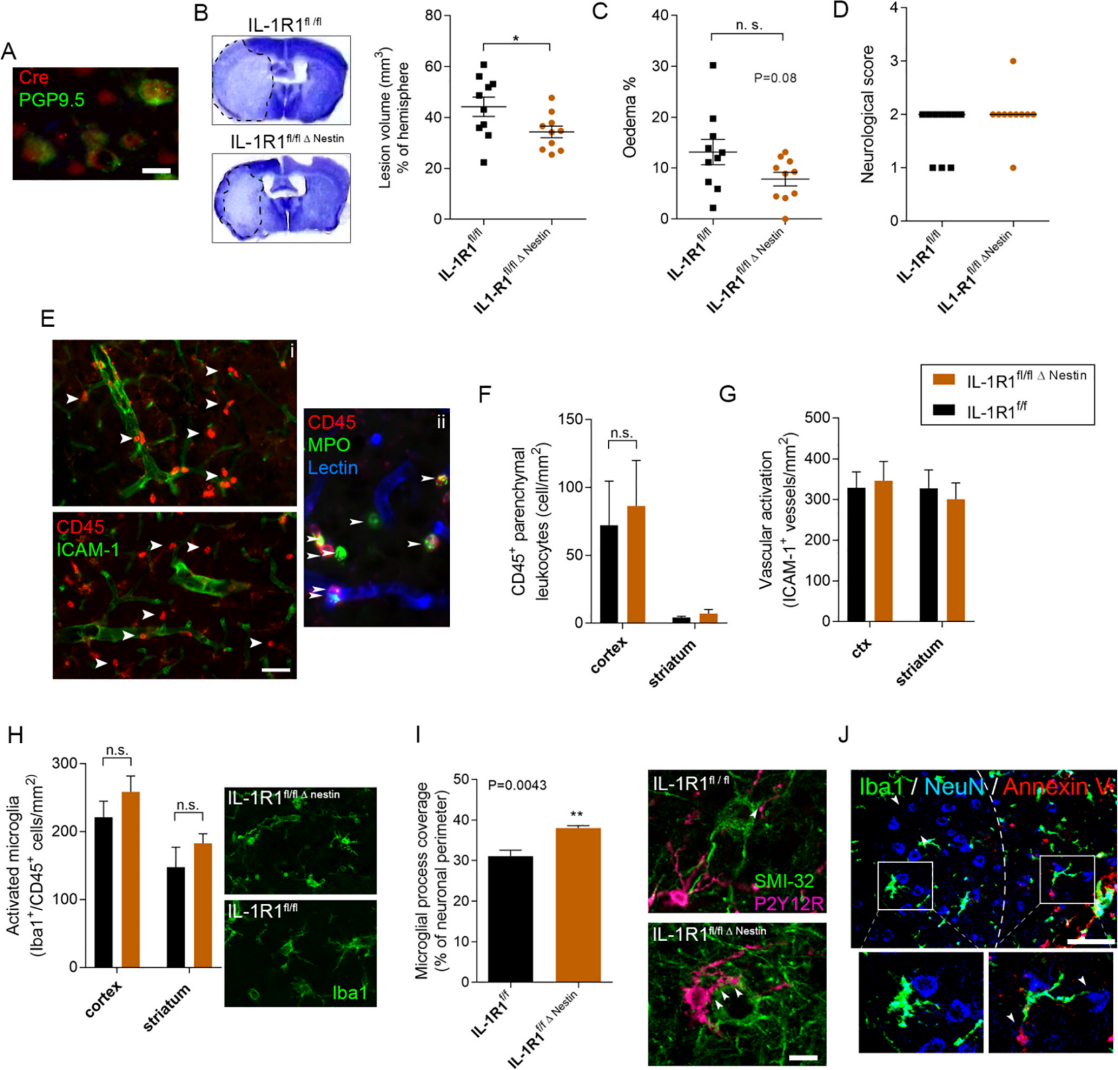
IL-1R1^fl/fl Δ Nestin^ mice are protected against brain injury and alter microglia-neuron interactions. Immunostaining reveals neuronal expression of cre recombinase in IL-1R1^fl/fl Δ Nestin^ mice 48 h after tamoxifen administration (A). IL-1R1 deletion in neurons (IL-1R1^fl/fl Δ Nestin^ mice treated with tamoxifen) reduced infarct volume (B), but did not significantly alter brain oedema (C) or neurological outcome (D). Dashed line outlines the area of the infarct. Immunofluorescent assessment of ICAM-1 expression in brain microvessels and CD45-positive leukocytes (arrowheads) (E) reveals no changes in the recruitment of blood-borne cells (F) and vascular activation (G) in response to neuronal IL-1R1 deletion. The majority of CD45-positive leukocytes were neutrophils containing myloperoxidase (MPO, arrowheads) (Eii). No differences were seen in the number of activated (Iba1 + CD45_low_) microglia in the striatum, or in the cortex (shown in pictures) (H). In contrast, microglial process coverage of neurons (arrowheads) was increased in the absence of neuronal IL-1R1 (I). Microglia (Iba1, green) contact neurons (NeuN, blue) and Annexin V-positive cell debris (red) in the boundary zone of the infarct. Note the markedly reduced number of neurons beyond the Annexin V-positive zone (dashed line) (J). n = 10. ^*^p ≤ 0.05, ^**^p ≤ 0.01. All parametric data are expressed as means ± SEM, medians are shown in the case of neurological scores. Scale bars: A, I, 10 μm; E, J, 50 μm. (For interpretation of the references to colour in this figure legend, the reader is referred to the web version of this article.)

To genetically delete IL-1R1 effectively from most neurons we used the nestin promoter-driven Cre recombinase expression that is expressed in neurons and a subset of glial precursors. Using our tamoxifen induction protocol we found that Cre expression was mainly confined to neurons in IL-1R1^fl/fl Δ Nestin^ mice 48 h after tamoxifen administration (Fig. 4A). Experimental stroke in IL-1R1^fl/fl Δ Nestin^ mice resulted in significantly smaller (25% reduction, p = 0.0379) brain injury (Fig. 4B) compared to IL-1R1^fl/fl^ mice that received identical tamoxifen treatment. In spite of this marked reduction in infarct volume, only a non-significant trend of reduced brain oedema was observed (Fig. 4C), whereas BBB breakdown was not affected in these mice (data not shown) and no difference was observed in neurological outcome (Fig. 4D). To understand these findings, we assessed changes in vascular activation and neutrophil recruitment in IL-1R1^fl/fl Δ Nestin^ and IL-1R1^fl/fl^ mice 24 h after cerebral ischaemia, as above. In contrast to the effect of brain endothelial IL-1R1 deletion, no changes in ICAM-1-positive blood vessels or the total number of CD45-positive leukocytes or neutrophils were found in the brain (Fig. 4E–G). The majority of CD45-positive leukocytes were neutrophils containing myeloperoxidase (Fig. 4E ii). The numbers of activated microglia were also not different (Fig. 4H), suggesting that an absence of IL-1R1 signalling in the brain of IL-1R1^fl/fl Δ Nestin^ mice does not influence vascular and microglial inflammatory actions in response to ischaemic injury. Since our previous research has revealed a key role for microglia in protecting injured neurons by shaping neuronal network activity and excitotoxicity, we assessed microglial process coverage of neurons as an indicator of altered neuronal activity and microglia-neuron interactions (Szalay et al., 2016). Microglial process coverage was significantly increased (by 22.29%, p = 0.0043) in the ischaemic penumbra of IL-1R1^fl/fl Δ Nestin^ mice (Fig. 4I), where microglia contacted both NeuN-positive neurons and Annexin V-positive cells beyond the zone of viable neurons (Fig. 4J). This suggested that neuronal IL-1R1 signalling may alter neuronal activity and /or protective microglia neuron interactions.

### 3.4 Brain endothelial, but not neuronal IL-1R1 deletion improves early perfusion deficits after cerebral ischaemia

**Fig. 5:**
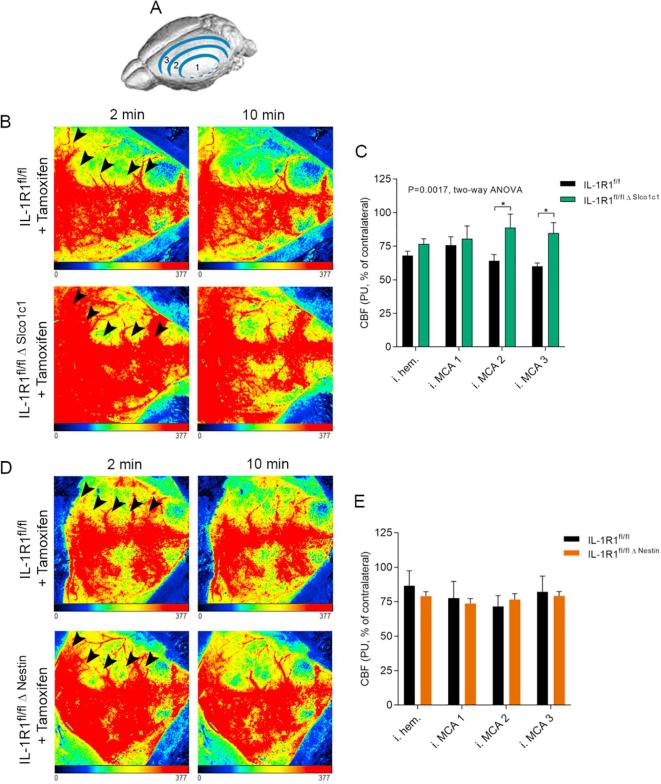
Endothelial IL-1R1 deletion reduces early perfusion deficits after cerebral ischaemia and reperfusion. Cortical perfusion was assessed by laser speckle contrast imaging (LSCI) in three adjacent zones (MCA1-MCA3) centred around the primary MCA area towards the midline and rostro-caudal cortical areas (A). IL-1R1^fl/fl Δ Slco1c1^ mice show better blood flow recovery at 30 min after the induction of reperfusion compared to IL-1R1^fl/fl^ mice, which is most apparent in zones MCA2 and 3 (arrowheads) during the 10 min measurement period. Note that cortical blood flow remains relatively uniform between the 2 min and 10 min representative time points. Quantitative analysis reveals significantly higher cerebral perfusion in the ipsilateral hemisphere (p ≤ 0.01, two-way ANOVA, all ROIs included) with Tukey’s post hoc multiple comparisons showing differences in the MCA2 and MCA3 zones (p ≤ 0.05, n = 5). LSCI maps after cerebral ischaemia and reperfusion show no difference between IL and 1R1^fl/fl Δ Nestin^ mice compared to IL-1R1^fl/fl^ mice (D) and quantitative analysis shows no significant changes in cortical blood flow (E). ^*^p ≤ 0.05, All data are expressed as means ± SEM.

To investigate whether site-specific IL-1 actions influenced brain perfusion early enough to influence the evolution of the infarct, we assessed cerebral perfusion changes 30 min after the induction of reperfusion in three adjacent regions of interest (ROI; Fig. 5A). In the absence of endothelial IL-1R1, a significantly smaller perfusion deficit was observed in the ipsilateral hemisphere at the MCA area [p = 0.0017, F (1, 32) = 11.74], which was apparent at both MCA2 and MCA3 ROIs (95% CI: 0.4004 to 49.09, p = 0.0451; 95% CI: 0.4911 to 49.18, p = 0.0440, respectively) compared to control mice (Fig. 5B and C). This phenomenon was less obvious in the lateral cortical areas (core of primary MCA territory) and was more apparent in the midline and rostral-caudal zones with more collateral supply (Fig. 5B, MCA2, MCA3 ROIs on Fig. 5C). We also assessed cerebral perfusion changes in IL-1R1^fl/fl Δ Nestin^ mice. In contrast to our findings after endothelial IL-1R1 deletion, no differences were observed between IL and 1R1^fl/fl Δ Nestin^ and IL-1R1^fl/fl^ mice (Fig. 5D and E), suggesting that the absence of IL-1R1 in neurons has no major impact on cerebral perfusion.

### 3.5 Deletion of IL-1R1 from cholinergic neurons results in smaller infarct size, smaller brain oedema and improved functional outcome

Our findings in IL-1R1^fl/fl Δ Nestin^ mice suggest the existence of parallel, endothelium-independent neuron specific actions of IL-1R1 in ischaemic brain injury. Therefore, we investigated the effect of IL-1R1 deletion directly in choline-acetyltransferase (ChAT) positive cholinergic cells. Forebrain cholinergic neurons play a fundamental role in controlling the CNS, establish widespread innervation in the forebrain and are implicated in cognitive decline and several neurodegenerative diseases. In addition, brainstem cholinergic neurons control various physiological functions and regulate general immune response via the vagus nerve (Hoover, 2017, Mufson et al., 2008, Duris et al., 2017).

**Fig. 6:**
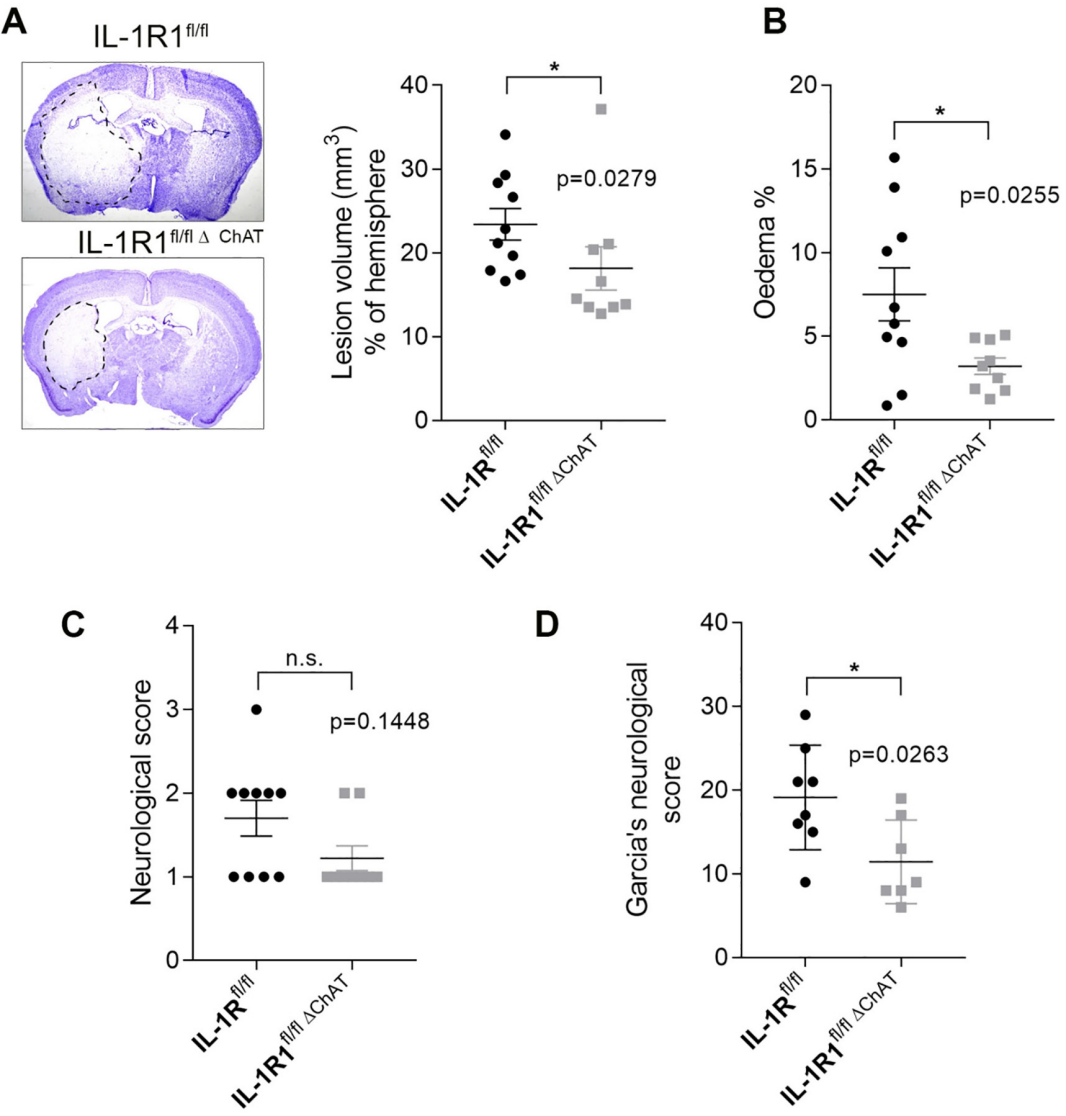
Deletion of IL-1R1 in cholinergic cells reduces brain injury, brain oedema and improves functional outcome after stroke. Deletion of IL-1R1 from cholinergic neurons (IL-1R1^fl/fl Δ ChAT^ mice) results in reduced infarct size (A) and brain oedema (B). A non-significant trend for improved sensory-motor function is seen in IL-1R1^fl/fl Δ ChAT^ mice (C), whilst functional outcome is significantly improved in these animals based on Garcia’s composite neurological score. n = 10 (IL-1R1^fl/fl^) and n = 9 (IL-1R1^fl/fl Δ ChAT^) (D). ^*^p ≤ 0.05, All parametric data are expressed as means ± SEM, medians are shown in the case of neurological scores.

There was a significant reduction in infarct size (by 22.4%, p = 0.0279) and brain oedema (by 57.2%, p = 0.0255) in IL-1R1^fl/fl Δ ChAT^ mice when compared to IL-1R1^fl/fl^ mice (Fig. 6A and B). Deletion of IL-1R1 in cholinergic cells also improved functional outcome as assessed by Garcia’s composite neurological score (Fig. 6D, p = 0.0263), whilst a non-significant trend for improved sensory-motor function was observed by using the Bederson score (Fig. 6C).

### 3.6 Removal of IL-1R1 signalling ubiquitously or from platelets and myeloid cells does not influence brain injury after cerebral ischaemia

**Fig. 7:**
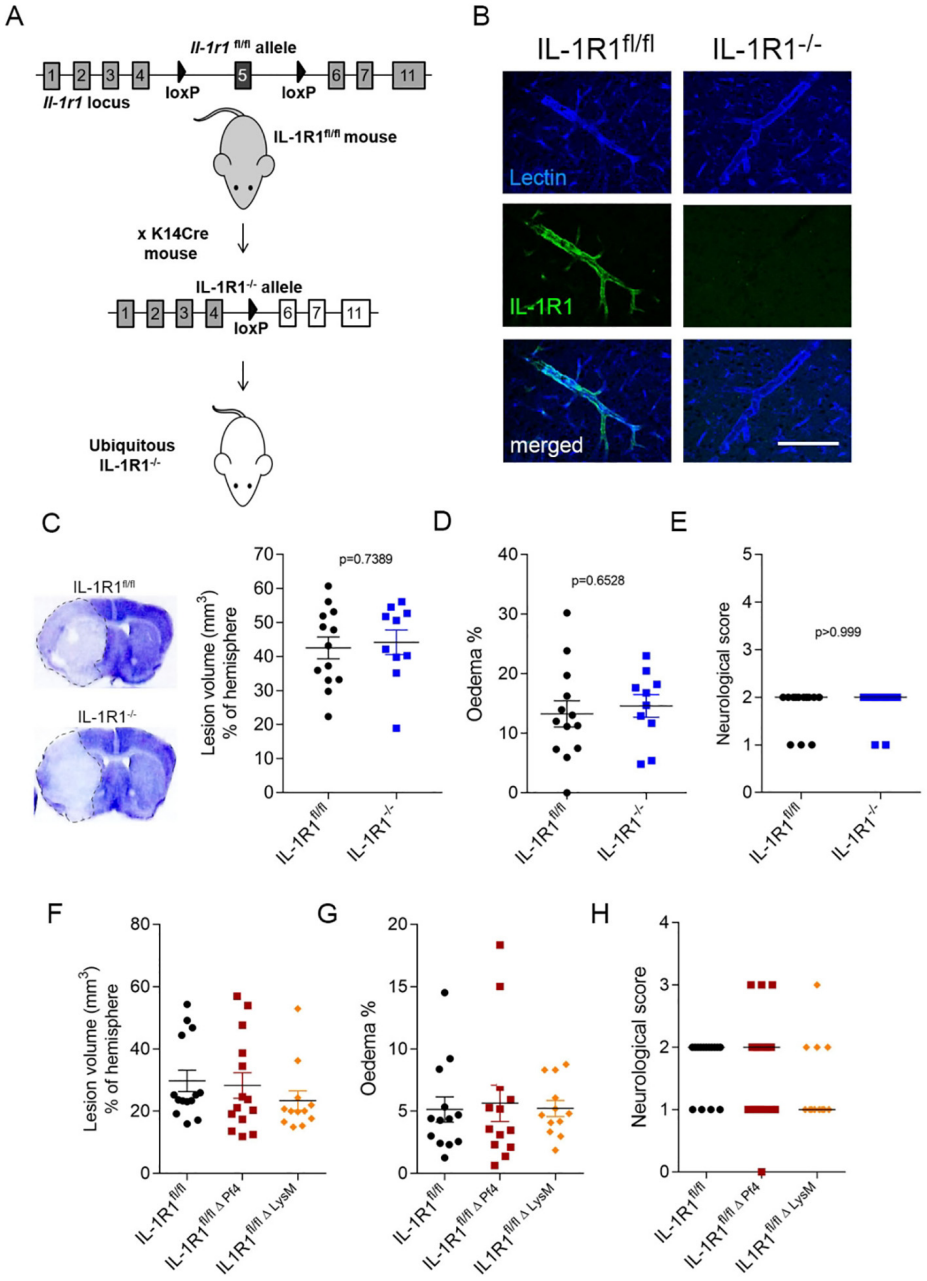
Ubiquitous and haematopoietic deletion of IL-1R1 do not influence outcome after cerebral ischaemia. IL-1R1^−/−^ mice (ubiquitous IL-1R1 deletion) were generated by crossing IL-1R1^fl/fl^ mice with mice expressing K14 Cre (A). IL-1R1^−/−^ mice show deletion of IL-1R1 protein expression in blood vessels labelled with tomato lectin (B). Infarct size (C), brain oedema (D) and neurological outcome (E) are not different in IL-1R1^−/−^ mice compared to IL-1R1^fl/fl^ mice. Deletion of IL-1R1 from platelets (IL-1R1^fl/fl Δ PF4^ mice) or from myeloid cells (IL-1R1^fl/fl Δ LysM^ mice) does not influence infarct size (F), brain oedema (G), and neurological scores (H) after experimental stroke. n = 13 (IL-1R1^fl/fl^), 10 (IL-1R1^−/−^) in B–D; n = 14 (IL-1R1^fl/fl^), 13 (IL-1R1^fl/fl Δ PF4^) and 12 (IL-1R1^fl/fl Δ LysM^). All parametric data are expressed as means ± SEM, medians are shown in the case of neurological scores.

We chose to flox exon 5 in the il-1r1 locus to effectively eliminate all IL-1R1 isoforms in order to generate a new line of IL-1R1^fl/fl^ mice where the receptor is fully eliminated (Fig. 7A). Ubiquitous deletion of IL-1R1 was confirmed with PCR from tail samples and a lack of IL-1R1 immunoreactivity in the brain (Fig. 7B). In spite of the complete absence of functional IL-1R1 in these mice and the marked protective effect of endothelial and neuronal IL-1R1 deletion, ubiquitous IL-1R1 KO mice subjected to cerebral ischaemia showed no difference in infarct size (Fig. 7C), oedema (Fig. 7D) and neurological outcome (Fig. 7E) compared to control WT (IL-1R1^fl/fl^) mice. To investigate whether IL-1R1 signalling in blood-borne cells could explain the lack of protection in ubiquitous IL-1R1 KO mice, but significantly reduced brain injury after brain endothelial and neuronal IL-1R1 deletion, we deleted IL-1R1 from platelets and myeloid cells. Constitutive deletion of IL-1R1 in platelets or myeloid cells by crossing IL-1R1^fl/fl^ mice with PF4-Cre mice and LysM-Cre mice, respectively, had no significant impact on brain injury and other parameters measured (Fig. 7F–H). Thus, endothelial and IL-1R1 in cholinergic neurons contributes to brain injury independently of IL-1 actions on platelets and myeloid cells.

## 4 Discussion

In this study we have identified the cerebrovascular endothelium and cholinergic neurons as major targets for IL-1 action after ischaemic stroke. Specific deletion of the IL-1R1 in brain endothelial cells reduced infarct volume and other outcome measures (i.e. BBB disruption, neurological deficit) induced by transient MCAo to a level comparable to that seen with IL-1Ra treatment in a number of previous studies (Maysami et al., 2016, McCann et al., 2016). We show for the first time that brain endothelial IL-1 actions have a robust and very early impact on cortical perfusion after brain injury, which is associated with facilitation of vascular activation and leukocyte infiltration. In contrast, neuronal IL-1R1 deletion reduced infarct size whilst having no influence on cerebral perfusion changes after stroke. This confirms IL-1 actions on the cerebrovascular endothelium and neurons as key events in mechanisms of IL-1-driven inflammation that depend on different mechanisms and potentially different time windows in response to stroke.

Brain endothelial cells are activated by IL-1 after stroke via binding of IL-1R1, leading to the upregulation of ICAM-1, VCAM-1 and P-Selectin expression, as well as various chemokines, resulting in neutrophil adhesion and infiltration (Thornton et al., 2010). We have demonstrated previously *in vitro* that IL-1 leads to the infiltration of neutrophils that acquire a neurotoxic phenotype (Allen et al., 2012). We now show *in vivo* that mice lacking IL-1R1 on brain endothelial cells exhibit reduced cerebrovascular expression of ICAM-1 and VCAM-1 that is accompanied by a marked reduction in the number of neutrophils infiltrating the brain in response to stroke, supporting our previous *in vitro* findings. Neutrophils are amongst the first cells to infiltrate the brain during cerebral ischaemia, amplifying cerebral inflammatory responses that exacerbate further BBB disruption, cerebral oedema and brain injury, leading to larger infarcts (Jin et al., 2010, Segel et al., 2011). Due to the key roles of neutrophils in ischaemic brain injury, it is not surprising they are of great interest as therapeutic targets (Jickling et al., 2015), and IL-1 acting on the brain endothelium to attract neutrophils and their neurotoxic intracellular content could potentially be a key mechanism. It is currently unclear whether neutrophil-dependent mechanisms contribute to changes in cerebral perfusion and/or BBB breakdown within the first hours after cerebral ischaemia. We have shown previously that IL-1-mediated systemic inflammatory mechanisms involve neutrophils, platelets and also stimulate endothelin-1 expression (Thornton et al., 2010, Denes et al., 2007, Murray et al., 2014), which could alter cerebral perfusion. Thus, IL-1 actions on the cerebrovascular endothelium could interact with several other IL-1 dependent (and independent) inflammatory processes in the mechanisms of brain injury. The surprising finding that the absence of functional IL-1R1 signalling in brain endothelial cells profoundly determines cerebral perfusion within the first hours – a therapeutically critical time window after stroke - suggests that timely blockade of IL-1 actions could have diverse beneficial effects on blood flow recovery and the associated inflammatory response that collectively determine functional outcome. Importantly, these effects of IL-1 on brain endothelial cells are potentially modifiable without the need for direct CNS actions of a drug, opening up the possibility of targeted anti-IL-1 therapies (e.g. neutralising antibodies) that would not typically show brain penetration.

We also observed microglial cell activation in the peri-infarct area but this activation was not affected by deletion of IL-1R1 in the brain endothelial cells or in the absence of neuronal IL-1 signalling. As well as changes to activation state due to cerebral injury, microglia also proliferate when activated (Weinstein et al., 2010). We saw no difference in the numbers of microglia between any of the treatment groups in the peri-infarct zone, which could be due to the short (24 h) survival times used in this study (Denes et al., 2007).

In contrast, deletion of IL-1R1 in IL-1R1^fl/fl Δ Nestin^ mice altered microglial process coverage of neurons, which indicates changes in microglia-neuron interactions after stroke. We have demonstrated recently that microglia are key contributors to neuronal network activity changes in the injured brain *in vivo* and an absence of microglia leads to markedly augmented neuronal injury after stroke (Szalay et al., 2016). This is likely due in part to an early protective action of microglia against excitotoxicity in the evolving penumbra (Szalay et al., 2016), which is associated with increased microglial process coverage of neurons that increases with higher level of neuronal activity. These results are consistent with observations of microglial actions on excitotoxicity using repetitive supramaximal stimulation and the effect of IL-1 on microglial process convergence toward neuronal axons in brain slices (Kato et al., 2016, Eyo et al., 2016). Increased microglial process coverage after neuronal, but not endothelial deletion of IL-1R1, could be linked to improved microglial control of neuronal activity, which should be investigated in detail in further studies.

To investigate the neurochemical phenotype of neurons that may mediate detrimental IL-1 effects via IL-1R1 signalling in the area of the brain supplied by the MCA, we chose to selectively eliminate IL-1R1 from cholinergic neurons. Cholinergic cells in the basal forebrain and the brainstem give rise to widespread innervation of the striatum (Dautan et al., 2014, Zaborszky et al., 2015) and the cerebral cortex. Besides the strong associations of the cholinergic system with attention, memory and cognitive function (Eckenstein et al., 1988, Rye et al., 1984, Yu and Dayan, 2005, Hasselmo and Bower, 1993, Rasmusson and Dykes, 1988, Jones, 1993), dysfunction of cholinergic neurons is linked with a wide array of neurodegenerative disorders (Tata et al., 2014). Atrophy of the basal forebrain cholinergic system is associated with cognitive impairment (Kilimann et al., 2017) and precedes Alzheimer’s disease pathology (Schmitz and Nathan Spreng, 2016). The mechanisms through which IL-1R1 signalling in cholinergic cells could mediate brain injury after experimental stoke are presently unclear. However, interactions between cholinergic pathways and the immune system are well-documented (Maurer and Williams, 2017). For example, α7 nicotinic acetylcholine receptors in astrocytes (Patel et al., 2017) and microglia (Suzuki et al., 2006) mediate anti-inflammatory effects including reduced release of TNFα and protect dopaminergic neurons against degeneration. In addition, systemic inflammatory responses are under the control of the cholinergic anti-inflammatory pathway (Rosas-Ballina and Tracey, 2009) supplied by connections of the vagal nerve. Activation of vagal neurons leads to smaller brain injury after experimental stroke (Jiang et al., 2014, Ay et al., 2009, Sun et al., 2012), whilst disruption to this pathway is linked with exacerbated brain injury in both ischaemic (Han et al., 2014b, Han et al., 2014a) and haemorrhagic (Krafft et al., 2012) stroke. Although IL-1 is known to regulate acetylcholinesterase production and activity of cholinergic neurons (Li et al., 2000), our data implicate for the first time that functional IL-1R1 in cholinergic neurons directly contribute to brain injury and brain oedema in an experimental model of brain injury. The important role of cholinergic innervation in shaping neuronal network activity throughout the brain, the sensitivity of cholinergic neurons to inflammation-mediated injury, or the role of ACh as a modulator of inflammation could explain the profound effect of cholinergic IL-1R1 deletion on stroke outcome. In fact, cholinergic neurons have been shown to be vulnerable to excitotoxicity and inflammation, which may also be indicated by changes in ACh levels or cholinergic receptors described in several neurodegenerative disorders (Weiss et al., 1994, McKinney and Jacksonville, 2005). Thus, IL-1-mediated actions could promote the dysfunction of cholinergic neurons via different forms of injury including increased excitoxicity and/or lead to impaired regulation of central and peripheral inflammatory processes by shaping the activity of the forebrain cholinergic system (Rosas-Ballina and Tracey, 2009, Allan and Rothwell, 2001), which will need to be investigated in further studies.

It is widely recognised that peripheral inflammatory disorders predispose to, or exacerbate, cardiovascular (Grundy et al., 1999) and cerebrovascular diseases (Catto and Grant, 1995). Experimental exacerbation of ischaemic brain injury by systemic infection and CNS pathology, induced by atherosclerosis, are mediated by peripheral IL-1 (Thornton et al., 2010, Denes et al., 2007, Denes et al., 2012). The cell targets and mechanisms of these effects are unknown, but the brain endothelium is a key candidate, since IL-1 penetrates the brain at very low levels (Banks et al., 1995). Circulating concentrations of IL-1 never reach high levels apart from in severe infection (Dinarello, 1996) but circulating immune cells may deliver IL-1 to the cerebrovasculature (Thornton et al., 2010), where IL-1R1 expression in the brain is highest (Konsman et al., 2004). We show, however, that although both platelets and myeloid cells produce IL-1 and contribute to brain injury after cerebral ischaemia (Iadecola and Anrather, 2011, Nieswandt et al., 2011), IL-1 actions via IL-1R1 in these cells are not required for these effects.

Previous research found that mice lacking IL-1R1 had comparable infarct volumes to wild-type (WT) animals (Touzani et al., 2002). Since in these IL-1R1 KO mice the expression of a truncated form of IL-1R1 could not be excluded (Qian et al., 2012), when generating our IL-1R1^fl/fl^ mice, we chose to flox exon 5 in the il-1r1 locus to effectively eliminate all IL-1R1 isoforms (Abdulaal et al., 2016). This new ubiquitous deletion of IL-1R1 did not significantly influence brain injury after stroke in the present experimental model, similar to what was found previously in other IL-1R1 KO mouse strains (Diem et al., 2003). It is possible therefore that a complete absence of IL-1R1 signalling could involve presently unknown compensatory effects and/or that beneficial actions via IL-1R1 might exist in the periphery or on other cells in the brain. We have not been able to explore such compensatory or potentially beneficial effects of IL-1 signalling in the present study, which we recognise as a limitation, and something that is the focus of ongoing future work. The protective effects of systemic IL-1Ra in different models of brain injury are widely recognised (Maysami et al., 2016), therefore further understanding of the cell-specific actions of IL-1 will need to be better understood to achieve a maximal potential for the therapeutic blockade of IL-1 actions in brain diseases.

**Fig. 8:**
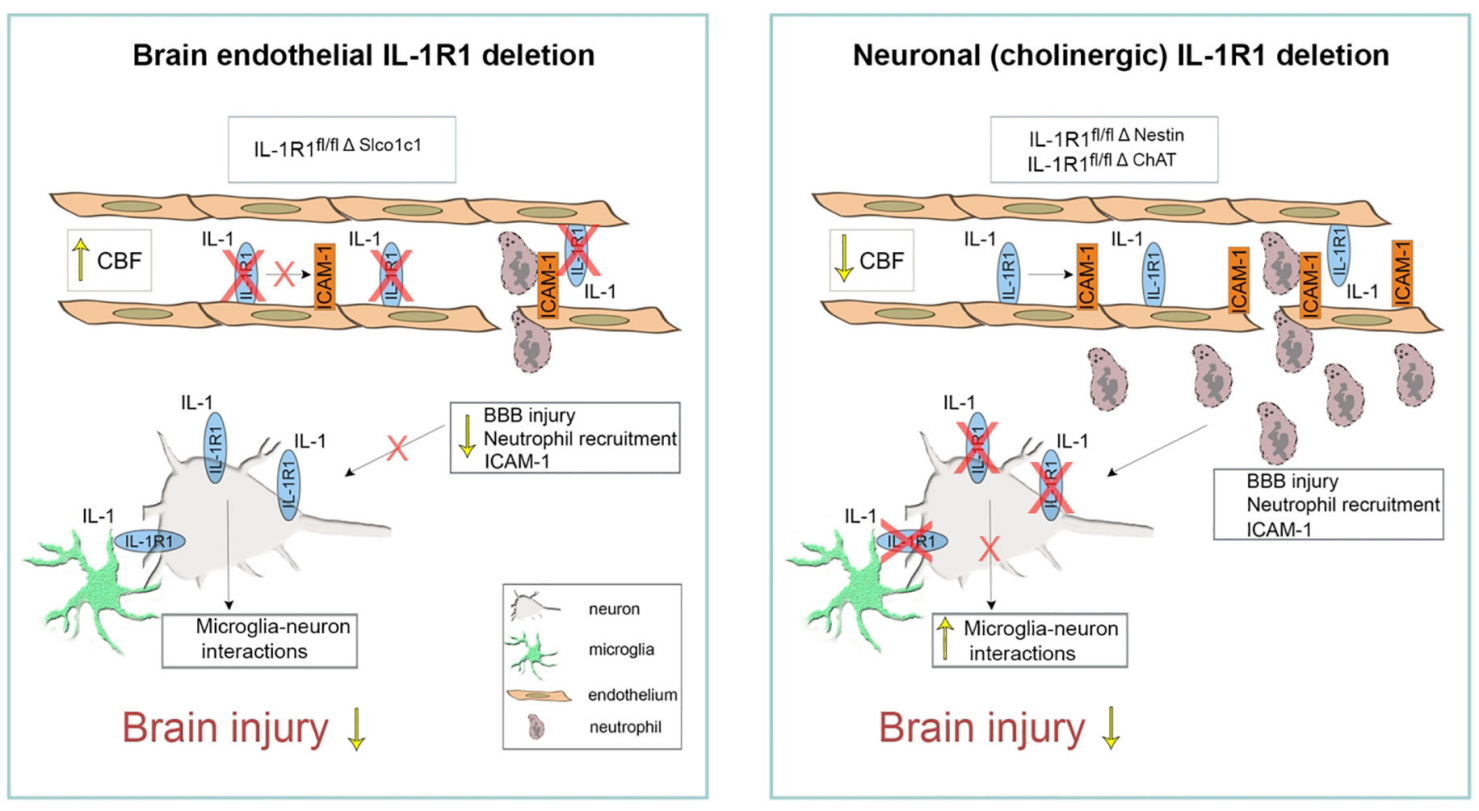
Summary of the distinct mechanisms of IL-1 actions on ischaemic brain injury targeting either the cerebrovascular endothelium or neurons. Our data show that both the cerebrovascular endothelium and neurons are primary targets of detrimental IL-1 actions mediating brain injury via two distinct mechanisms. Endothelial IL-1R1 deletion results in markedly decreased infarct size, BBB injury and better neurological outcome 24 h after stroke. It is also associated with decreased vascular activation and neutrophil recruitment. Interestingly, an improved cerebral perfusion can be seen in these mice 30 min after reperfusion indicating that IL-1 has early detrimental actions on cerebral blood flow acting through the vascular endothelium. Neuronal IL-1R1 deletion is also protective regarding infarct size, however BBB injury, vascular activation, neutrophil recruitment and early cortical perfusion are unaffected by the absence of IL-1R1 in cholinergic cells or neurons. However, increased microglial process coverage can be detected around neurons in the penumbra suggesting altered microglia-neuron interactions.

In conclusion, brain endothelial and neuronal (cholinergic) IL-1R1 are key contributors to the detrimental actions of IL-1 in the brain after stroke (Fig. 8). Cell-specific targeting of IL-1R1 in the brain could have major therapeutic benefit in stroke and other cerebrovascular disease by allowing more selective targeting, improving efficacy and reducing any potential side effects.

## Funding

This work was supported with funding from the British Heart Foundation (grant ref: PG/13/8/29989 to SA, EP and NJR), the Hungarian Brain Research Program [KTIA_13_NAP-A-I/2 (AD) and 2017-1.2.1-NKP-2017-00002 (GN)], National Research, Development and Innovation Office, NN 125643 (GN), the ‘Momentum’ Program of the Hungarian Academy of Sciences (AD) and ERC-CoG 724994 (AD). The generation of the IL‐1R1^fl/fl^ mice was funded by FP7/EU Project MUGEN (MUGEN LSHG‐CT‐2005‐005203) to WM and the Medical Research Council (G0801296) to SA, EP and NJR.
